# A Review of Village Ecosystem Vulnerability and Resilience: Implications for the Rocky Desertification Control

**DOI:** 10.3390/ijerph19116664

**Published:** 2022-05-30

**Authors:** Jiuhan Tang, Kangning Xiong, Yue Chen, Qi Wang, Bin Ying, Jiayi Zhou

**Affiliations:** 1 School of Karst Science, Guizhou Normal University, Guiyang 550001, China; karst_tangjh@163.com (J.T.); cychenyy@163.com (Y.C.); waldowangqi@163.com (Q.W.); yingbin@gznu.edu.cn (B.Y.); zhoujiayi2225@163.com (J.Z.); 2 State Engineering Technology Institute for Karst Desertification Control, Guiyang 550001, China

**Keywords:** village ecosystem, vulnerability, resilience, review, rocky desertification

## Abstract

Under the background of global environmental change, a huge impact has been made on the village ecosystem, which leads to disorder of structure and function of the village ecosystem. The current management measures of the village have failed in allowing the village to achieve sustainable development. Research on the vulnerability and resilience of the village ecosystem is helpful in regards to the ecological restoration of the village. The research status and progress in regards to the vulnerability and resilience of the village ecosystem are not clear, and the summary of research results and problems is insufficient. Based on 87 related literatures, this paper focuses on the current status and progress of village ecosystem vulnerability and resilience research, and reveals the current research results and shortcomings of village ecosystem vulnerability and resilience. We found that: (1) the research on vulnerability and resilience of the village ecosystem is on the rise; (2) the research mainly focuses on the index system, monitoring and assessment, mechanism research and strategy research. The monitoring and assessment research is the most prominent, which mainly discusses the research methods, the vulnerability and the resilience of the village ecosystem; (3) the study area is mainly concentrated in Asia, North America and Africa. Research institutions are mainly institutions of higher learning and research institutes (centers). Finally, this paper finds that major scientific and technical studies such as the construction of indicator systems and the study of governance strategies in the study of vulnerability and resilience of village ecosystems are lagging behind. In future research, we should deepen the research on the concept and connotation of vulnerability and resilience. We must establish a scientific and reasonable research framework for vulnerability and resilience of the village ecosystem. We should also strengthen and improve the index system of vulnerability and resilience of the village ecosystem. We should strengthen research on the impact mechanisms and governance strategies of vulnerability and resilience, and apply the research on vulnerability and resilience to the planning and governance of the village ecosystem.

## 1. Introduction

Ecosystems are affected by global change, extreme disasters occur frequently, greenhouse gas concentrations increase, biodiversity has been lost, water pollution and photochemical air pollution and other ecological and environmental problems are prominent. This leads to the damage of ecosystem function and the decline of self-regulation ability. The impact on both human society and the natural environment has been far-reaching [1,2]. With the development of urbanization, the waste of resources and environmental pollution further aggravate the ecosystem recession [3]. The interactive process of population growth and urbanization has had an important impact on the sustainable use of resources, leading to overexploitation of the ecosystem [4]. Advances in technology have increased human disruption of global ecosystems and the services they provide [5]. Sustained economic growth is likely to exceed the ecological threshold of ecological society and destroy the ecosystem services on which humans and all other species depend [6]. Studies have shown that 60% of the global ecosystem services have been degraded due to population growth and economic growth [7]. In addition, the increased frequency of extreme weather events has also affected the supply of ecosystem services; for example, heavy rainfall can limit potential recreational opportunities in parks and has a serious impact on the supply of ecosystem services [8]. Extreme weather events, such as droughts, heat waves, storms and cold waves, have led to inadequate water availability and have affected crop yields, and thus they affect the supply of ecosystem services [9,10]. Therefore, under the impact of human social development and ecological degradation, the protection of the ecosystem has been put on the international political agenda [11].

The village ecosystem is a complex system which is based on villages, with rural population as the core, associated organisms as the main biological communities, building facilities as the important habitat environment, including economy, society and ecology, and encompassing production and living activities of the rural population [12,13]. It has the functions of carrying population, providing products, improving the environment, inheriting culture, sightseeing tourism, popular science education and providing for aged urban residents [14]. The vulnerability of the village ecosystem is a natural attribute. It is manifested in the weak ability to withstand external interference, and it is difficult to restore to a stable and balanced state after the impact. With the development of the social economy, against the background of globalization, industrialization and urbanization, the global countryside is undergoing a holistic restructuring. This is accompanied by environmental pollution, ecological damage, natural disasters, waste of resources, population loss and other problems [14,15]. The advancement of urbanization leads to the outflow of the rural population, and the self-development ability and adaptability of rural areas are constantly challenged. The high frequency of climatic and geological hazards exacerbates the instability and vulnerability of development in rural areas; the resulting rural decline has become a global problem [16,17,18].

Village ecosystems provide most of the world’s food and natural resources, carbon sequestration, water filtration and wildlife habitat, as well as maintaining supplies of food, energy and freshwater products for rural and urban populations. Coastal wetlands also provide storm surge protection [19,20,21,22]. However, villages are becoming vulnerable due to various natural and human factors [23,24,25,26]. Most rural livelihoods are directly dependent on natural resources. Irrational exploitation and overexploitation of nature has led to a series of ecological degradation problems such as desertification, rock desertification and salinization of the land. For developing countries, villages are still the main bottleneck restricting social and economic development. Especially in recent decades, the inland rural areas have undergone significant economic and social changes. The economic and demographic transition and the increasing marginalization associated with population decline have led to rising unemployment, the emigration of economically active groups and population ageing [27]. In addition, the coastal countryside is threatened by sea level rise, storm surges and inland flooding [19]. In order to reduce the vulnerability of the village ecosystem and enhance its anti-interference ability, adaptability and recovery ability, some scholars have introduced resilience into the study of village ecosystems [28,29]. Village ecosystem resilience means that when the village ecosystem is disturbed and impacted, the system has the ability to adjust its structure to cope with the disturbance and make the system reach a new stable state equilibrium along a new recovery path [15]. However, resilience and vulnerability are not simply opposites and subordinate, but are interlinked and can be positively or negatively correlated [30,31,32]. For example, karst rocky desertification area, because of its special human activities, geographical environment and lithological characteristics, leads to soil, hydrology, vegetation and human environment fragility, forming a fragile ecosystem similar to the edge of the desert [33,34,35]. In order to improve this situation, human beings should deal with the relationship between man and environment, economic development and ecological protection. With the concept of sustainable development, based on the characteristics of the ecological environment of rocky desertification, with water storage, soil management and afforestation as the core, we could develop integrated management techniques for different rocky desertification intensity zones, and we propose an integrated management technology model suitable for karst rocky desertification [36] in order to contribute to the overall improvement of the ecosystem.

As an indispensable part of the global ecosystem, the village ecosystem is of great significance to global sustainable development. The study of village vulnerability and resilience has received increasing attention from scholars from all walks of life. However, the progress and shortcomings of current research on vulnerability and resilience of village ecosystems are unknown. Therefore, this paper summarizes the current research results of vulnerability and resilience of the village ecosystem. In addition, the key scientific and technological problems to be solved in future research in this field are prospected. This points out the direction for further research on the vulnerability and resilience of the village ecosystem in the future, in order to provide scientific and technological references for promoting the sustainable development of rocky desertification control villages.

## 2. Methods

In order to select scientific papers to quantitatively analyze the research dynamics of vulnerability and resilience of the village ecosystem, we conducted a literature search in the Web of Science Core Database (WOS) and the China National Knowledge Infrastructure (CNKI). The reason why we chose WOS core database and CNKI database is that they contain research in many fields such as natural science and social science, and the number of articles included is very large, so they can meet the needs of our research. The time range for our retrieval is the maximum time range, as at 31 December 2021. We used the following syntax for retrieval: TS = ((ecosystem AND vulnerability AND resilience) AND (village OR rural OR countryside OR settlement OR community)). A total of 861 articles were obtained. We identified and screened the obtained literatures at the level of title and abstract. We also defined three criteria for screening: (1) pay attention to villages; (2) pay attention to the human-environment coupling system; (3) focus on both vulnerability and resilience. The above three conditions must all have been met. For uncertain literature at the title and abstract level, we decided whether to use the literature as a sample by reading the full text. Finally, we identified 87 literatures, including 6 doctoral dissertations, 9 master’s dissertations and 72 journal articles. We analyzed the number of papers published each year, the institutions that published the papers, the types of research papers and the research sites of the papers on the vulnerability and resilience of village ecosystems.

## 3. Results

### 3.1. Annual Distribution of Literature

**Figure 1:**
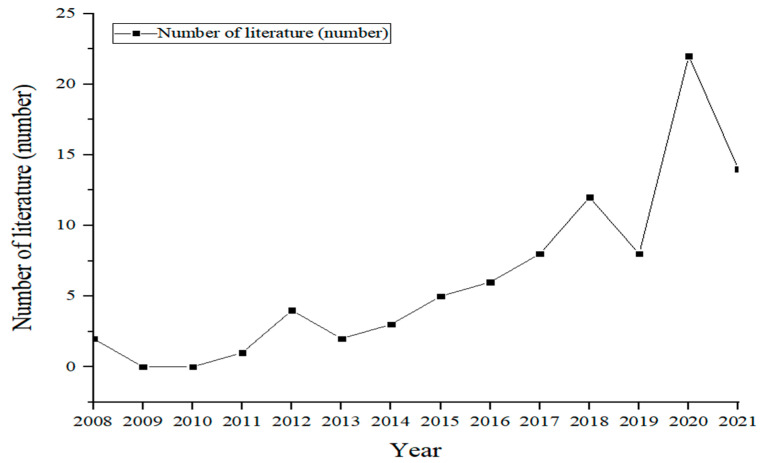
Annual distribution of research documents about vulnerability and resilience of the village ecosystem.

The research on the vulnerability and resilience of the village ecosystem is generally on the rise, and can be roughly divided into three stages (Figure 1). In the first stage, from 2008 to 2013, the total number of documents did not exceed 9; this was the embryonic stage. The second stage was from 2013 to 2018, which is a slow growth period. In the third stage, from 2018 to 2021, there was a rapid growth trend, with an average annual literature count of more than 13.

### 3.2. Content Distribution of the Literature

**Figure 2:**
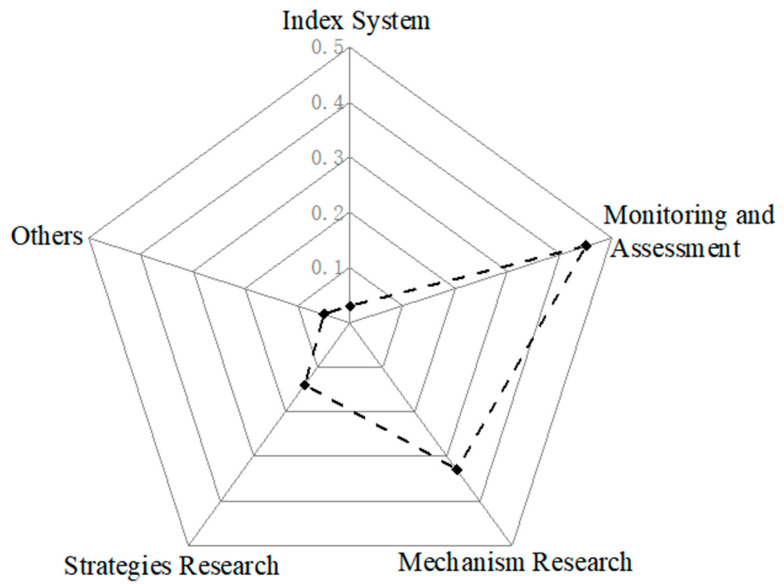
Literature by content.

According to the research content, all the literatures are classified and summarized according to the index system, monitoring and evaluation, mechanism research, strategy research and other related research. The index system accounts for 3%, monitoring and evaluation accounts for 45%, mechanism research accounts for 33%, strategy research accounts for 14% and other types of literature account for 5% of the total, as in Figure 2. According to the proportion of literature types, the research on vulnerability and resilience of the village ecosystem is mainly focused on monitoring and evaluation and mechanism research, and the index system and strategy research are still in the exploratory stage of development.

### 3.3. Distribution of Study Area of Literature

**Figure 3:**
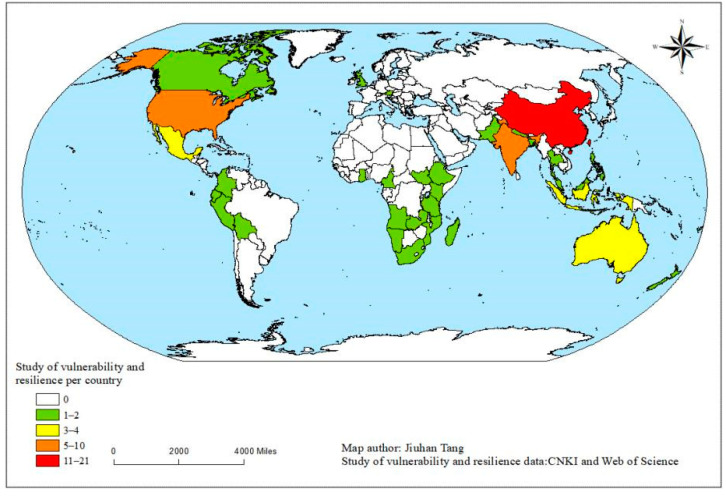
Distribution of study area.

According to the collation of the literature, we generated statistics and analysis on the study area of 87 literatures obtained. The results show that the study area of scientific literature on vulnerability and resilience of village ecosystems covers 38 countries. The majority of studies were concentrated in Asia (50%), North America (16%) and Africa (15%), with the largest number of studies conducted in China, with 21 articles, accounting for 23% of the total. India and the USA came second with 10 papers each, accounting for 11% of the total. This is followed by 3 or more articles in the literature for studies conducted in Indonesia, Australia and Mexico. Other countries have 1–2 articles in the literature (Figure 3).

### 3.4. Institution Distribution of the Literature

**Figure 4:**
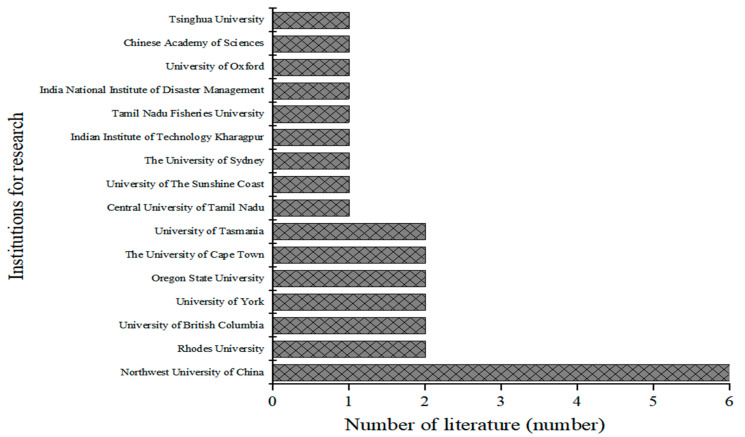
Literature by institution.

To interpret and analyze the units of publication on the vulnerability and resilience of village ecosystems, we used the affiliation of the first author of the literature as the basis. The research literature was published by research units from 25 countries, with the majority of the literature coming from China (22%), the USA (16%), India (11%) and Australia (10%). The authors’ affiliations are divided into four main types: higher education institutions, research institutes (center), governmental organizations and non-governmental organizations, which account for 71%, 23%, 4% and 2% of the total number of research institutions, respectively. The units involved in the relevant research are mainly universities and research institutes (centers). Major research units include Northwestern University in China, Rhodes University, University of British Columbia and Oregon State University (Figure 4). In most of these, the main units are ecological and environmental protection, disaster resource management, ecological and environmental higher education institutions and research institutes.

## 4. Discussion

### 4.1. Main Progress and Landmark Achievements

#### 4.1.1. Indicator System

(1) Based on the vulnerability characteristics and formation mechanisms of village ecosystems, a system of indicators for vulnerability evaluation in different regions was constructed.

The reasonableness of the indicator system is related to the credibility and accuracy of the evaluation results. At present, there are various types of indicator systems for vulnerability assessment of the village ecosystem, which are often constructed according to the particularity of the study area. Vegetation coverage, drought frequency, land use intensity, land desertification area, water resources, water quality, effective irrigation area, afforestation area and cultivated land area are the vulnerability indicators of the arid village [37]. In coastal villages, ecosystems are mainly affected by oceanic disturbances, such as sea-level rise, tsunamis, typhoons and coastal erosion. For coastal erosion ecosystems, the distance of farmers from the ocean, fishing location, coastal protection, vegetation and so on are targeted indicators [38]. In the semi-arid region of the Loess Plateau, drought disaster, forest cover, drinking water safety, rain and waterlogging disaster, wind and sand disaster, land cultivation, fertilizer application, population factors, social infrastructure and so on are the key indicators of the vulnerability of the rural human settlements system [39]. Agricultural facilities, the distance from the river bank, the use time of houses, building materials, disaster losses, emergency shelters, disaster knowledge, disaster warning system and other indicators are the indicators that distinguish the disaster village ecosystem from other fragile ecosystems [40]. However, fewer studies are related to rocky desertification village ecosystems. The vulnerability assessment indicator system of the village ecosystem under the rocky desertification control environment is still deficient, and it is necessary to construct the vulnerability assessment indicator system according to the characteristics of the natural ecology and socioeconomic activities of the rocky desertification control village ecosystem.

(2) Based on natural, social and economic vulnerability factors, a comprehensive evaluation Indicator system is constructed by integrating internal and external factors of the system.

The normative and unified ecological vulnerability assessment index system is the basis of quantitative analysis of ecological vulnerability; the selection of the ecological vulnerability assessment index directly affects the scientific evaluation results and regional development policy [41]. The single indicator system has strong pertinence and regionality, and can determine the key factors leading to regional environmental vulnerability according to regional characteristics [42]. However, the structure is simple and the content is not comprehensive. Village ecosystems are complex systems that include natural ecological and socioeconomic elements. Therefore, a single indicator system is not suitable for village ecosystem research. The common research is based on nature–society–economy and other aspects, combined with the pressure–state–response model, exposure–sensitivity–adaptability model and sensitivity–resilience–pressure model and other conceptual framework models to build a comprehensive village ecosystem vulnerability assessment indicator system. The scholars used the exposure–sensitivity–adaptability model to construct a comprehensive evaluation index system for village vulnerability from the socioeconomic and natural ecological environment perspectives [43,44]. Berrouet et al. constructed a comprehensive indicator system of village vulnerability assessment from the perspective of social economy and natural environment [45]. At present, there is no uniform standard for the index system established according to different conceptual models, and the accuracy and scientific rationality of the evaluation results need to be verified.

#### 4.1.2. Monitoring and Assessment

(1) Through qualitative analysis and quantitative evaluation methods, the vulnerability and resilience of ecosystem were evaluated.

The methods of vulnerability assessment are classified into qualitative analysis and quantitative assessment [46]. Qualitative analysis is based on the historical evolution of the system and the current state of in-depth study, based on various aspects of data and experience of the impact of system vulnerability and resilience factors on qualitative judgment. Qian Zhang qualitatively analyzed the social vulnerability of herdsmen in the Inner Mongolia desert grassland. The study found that the division of grassland into households and the introduction of the market mechanism increased the risk exposure of herdsmen. A series of grassland protection projects implemented by the government have caused many restrictions on herdsmen’s disaster response measures. It has led to a reduction in the ability of pastoralists to cope with climate change and increased social vulnerability [47]. Chelleri has qualitatively analyzed the relationship between community resilience and vulnerability in the southern highlands of Bolivia [48]. The quantitative evaluation method entails using the mathematical model analysis method to quantitatively evaluate the possible damage or impact of disturbance factors on the ecosystem through data information. The research on vulnerability and resilience involves many disciplines and fields. The quantitative evaluation method is to deconstruct the vulnerability and resilience into several components and evaluate them respectively, and then construct a function model to calculate the vulnerability and toughness indicator according to the relationship between the components. In addition, there are many types of research methods; with the development of research, the research methods on vulnerability and the resilience of the village ecosystem are more complex and diversified. These include analytic hierarchy process [49], entropy method [50] and principal component analysis [51]. Each of these research methods has its own advantages and disadvantages, and the practicability and defects of the evaluation methods have a greater impact on the evaluation results.

(2) Ecosystem vulnerability and resilience assessment is a system quality assessment with temporal and spatial characteristics, which can be divided into static assessment and dynamic assessment.

Static evaluation is used to evaluate the vulnerability and resilience of ecosystems at a specific time, and to compare the vulnerability and resilience of ecosystems in different regions. Ghosh (2021) et al. studied the current vulnerability of villages in flood-prone areas. It also compares the vulnerability of agricultural villages and forest-resource-dependent villages in a specific time [52]. The data selected by dynamic evaluation are continuous in time, and the data selected are generally multi-year data for the study period. It is mainly used to study the temporal and spatial trends of vulnerability and resilience in the same region, and can directly analyze the changes of regional ecological environment quality. The development and application of 3S technology provides powerful technical support for the dynamic research of geographical entities. Relevant scholars analyzed the temporal and spatial changes of village vulnerability in the Loess Plateau through the data of three time periods [26]. Peng et al. conducted a study on village resilience after vulnerability to seismic hazards, comparing differences in resilience in the first and second years after the disaster [53]. Static assessment is only for vulnerability and resilience status analysis; dynamic assessment can clarify the temporal and spatial changes of vulnerability and resilience, and can more intuitively reflect the changes of vulnerability and toughness.

(3) In the context of climate change, vulnerability is constantly changing, through the simulation of indicators to predict the future vulnerability of ecosystems.

Climate change is considered a serious threat to the earth, with global temperatures rising and extreme weather events occurring frequently. Climate change has a range of impacts on human health and agriculture, forests and water resources [54]. Small changes in daily weather phenomena such as humidity and temperature bring significant changes to the production and life of villages [55]. To effectively address future climate change vulnerability, Dasgupta et al. projected the future vulnerability of villages in the Himalayas based on preselected indicators of natural, human, financial and physical capital assets, based on exposure–sensitivity–adaptability, and based on the projections recommended that future policy interventions should target climate-sensitive sectors [56]. Jara et al. predicted the future socioecological vulnerability of Peruvian fishing communities and used the predicted results to propose adaptation options [57]. Most of the predictions are based on scenario simulation methods; the village ecosystem is affected by natural and human disturbance, and its changes are complex and diverse, so it is difficult to accurately predict the future vulnerability changes through scenario simulation methods.

#### 4.1.3. Mechanism Research

(1) Aiming at the problem that the cause of village ecosystem vulnerability is not clear, the formation mechanism of village ecosystem vulnerability is revealed by analyzing the interaction between ecosystem elements.

The human–land coupling ecosystem is composed of the natural ecological environment and human economic factors. Geological structure, geomorphological features, surface material composition, biodiversity and other factors are the material basis of the ecological environment [58]. Species diversity affects ecosystem stability and the sustainability of ecosystem functions and services [59]. In order to meet the needs of livelihood, obtain economic benefits and adapt to social and environmental changes, human beings constantly change the ecosystem [60,61]. The resulting greenhouse effect, water shortages, species extinction and other ecological and environmental problems exacerbate the vulnerability of ecosystems [62,63]. The formation mechanism of vulnerability is different under different natural environment and human activities. Villages in arid environments have poor diversity of vegetation types, high exposure and sensitivity due to water shortage. The lack of social capital, human capital and financial capital leads to the lack of adaptive capacity, which ultimately leads to the fragility of the village ecosystem [64]. Villages will also develop high sensitivity and low adaptability because of their remote location, inconvenient transportation and lack of infrastructure, lack of education and income of residents, and poor social network, resulting in fragile village ecosystems [65]. Generally speaking, the vulnerability of the village ecosystem is formed by the interaction of system elements. Ecosystems with poor natural conditions and disharmonious human–land relationships are prone to increase vulnerability, while ecosystems with superior natural conditions and harmonious human–land relationships will reduce vulnerability.

(2) Identifying the disturbance factors of ecosystem and revealing the driving factors of ecosystem vulnerability in different types of environmental villages.

Disturbance factors of the ecosystem include natural disturbance factors and artificial disturbance factors. Ecosystems under different natural conditions suffer different types of disturbances, and their vulnerability driving factors are also different. The increasing intensity and frequency of extreme events such as floods, droughts, tropical cyclones and storms caused by global climate change [66,67,68] have a huge impact on the ecosystem. Geological disasters such as earthquakes, landslides and debris flow also disturb and impact the ecosystem. Human disturbance is mainly reflected in the development of natural resources, the construction of infrastructure, unreasonable waste discharge and so on. The environmental and socioeconomic problems of villages caused by human activities, climate change and extreme events are undeniable and affect different regions in different ways. Rising sea levels and saltwater intrusion often limit agricultural development in coastal areas, thereby affecting rural livelihoods, exacerbating local economic hardship and creating unique vulnerabilities for coastal rural communities [69]. The riverbank erosion caused by the flood is the reason for the fragile village ecosystem. The flood leads to the reduction of arable land, the displacement of farmers, and food insecurity [70]. Unreasonable human activities, such as increased rainfall in the upper reaches of the river, poor agricultural practices and sand mining, will increase the exposure and sensitivity of villages in the middle and lower reaches of the river to river floods [71]. Meteorological disasters, such as flood and drought, are the driving factors for the formation of vulnerability of planting-dependent villages [24]. The dominant factors of village ecosystem vulnerability are different under different natural conditions; natural disturbances are mainly natural disasters, and manmade disturbances are mainly unreasonable human social and economic activities. It is of great significance to study the dominant factors of ecosystem vulnerability for alleviating the stress of external pressure and maintaining the sustainable development of the system.

#### 4.1.4. Strategy Research

(1) Based on the characteristics of vulnerability, the formation mechanism and its impact on the ecological environment, the ecological management strategies are put forward.

As population growth and other pressures on ecosystems increase, they will affect the ability to provide ecosystem services, so it is necessary to deal with the relationship between socioeconomic development and ecosystems. Before solving the problem of the vulnerability of the ecosystem, we first need to identify the objects of vulnerability and the mechanisms of vulnerability formation. Due to the differences in geographical environment and human social and economic activities, the characteristics of ecosystems are significantly different, and their vulnerability characteristics are also different, so it is necessary to put forward appropriate governance strategies according to different types of fragile ecosystems. Vulnerability research focuses on the structure and function of the system itself, assessing and predicting the likely impact of external stressors on the system. Its purpose is to maintain the sustainable development of the system, to reduce the adverse effects of external stress on the system and to provide the decision-making basis for the comprehensive management of the degraded ecosystem [72]. Vulnerability research is the basis of ecosystem governance and restoration. Due to the impact of climate change, vulnerability is significantly affected by low per capita income, poor agricultural climate conditions, aging of household heads and farm size. Therefore, it is necessary to develop special policy plans, expand farm size, improve farmers’ understanding of the driving factors and process of climate change, and increase rural income to achieve village restoration [73]. The livelihoods of rural household dependent on natural resources in the context of climate change are affected by climate change, natural disasters and livelihood strategies, which should be diversified, with enhanced education and the establishment of early warning systems for extreme weather [74]. In drought-prone areas, it is necessary to enhance farmers’ social, human, financial and material capabilities, publicize knowledge of extreme events such as drought, and strengthen infrastructure construction to improve farmers’ disaster resistance [75]. In view of the rocky desertification control village ecosystem, it is necessary to clarify the vulnerability factors and formulate ecosystem management strategies according to the influencing factors.

(2) Resilience research provides a new way of thinking about ecosystem governance and proposes targeted governance strategies based on the drivers of resilience in different types of ecosystems.

Resilience research is used to analyze the ability of the ecosystem to absorb and deal with disturbances and to restore itself to a sustainable state after being disturbed and impacted by natural or human factors. Recent scientific research shows that an increasing number of studies use resilience to analyze the sustainability dynamics of villages. The interdisciplinary analysis of villages covers various aspects such as human ecology, ecological economics, rural sociology and environmental studies, and discusses the internal and external factors that influence the sustainability dynamics of the village [76]. The current research on village resilience mainly focuses on resilience evaluation and resilience enhancement strategies. For example, Roy et al. put forward the management strategies of the coastal agroecosystem based on the resilience assessment of the coastal agroecosystem [77]. Lwin et al. quantitatively assessed the resilience of flood-prone villages in Myanmar’s Rowaddy River Delta through indicators, and proposed response strategies based on the driving factors of resilience [78]. Although research on resilience involves many types of village ecosystem, research on the resilience of the rocky desertification control village ecosystem is still rare, so it is necessary to learn from the existing research methods and means to strengthen the study of the resilience of the rocky desertification control village ecosystem.

### 4.2. Key Scientific Issues to Be Solved

#### 4.2.1. In View of the Lack of Research on the Combination of Vulnerability and Toughness of Karst Ecosystem, a Research Framework Suitable for the Combination of Vulnerability and Toughness of Karst Ecosystem Should Be Established

Vulnerability and resilience are themes in sustainable development research [32]. Although these two concepts have been widely used, the relationship between them has not yet reached a consensus [79]. At present, there are many studies on vulnerability and resilience which involve many disciplines and research fields, but most of them have a single research perspective and there is lack of a perfect and effective integrated research framework. If a better interface between vulnerability and resilience integration studies is to be achieved, the concepts and theoretical understanding framework of vulnerability and resilience need to be clarified, and the relationship between vulnerability and resilience of specific ecosystems needs to be clarified. A break with traditional research methods to develop new research framework models from a multi-disciplinary perspective is needed. At present, research on the vulnerability and toughness of karst is mostly concentrated in the field of ecological environment and water resources [80,81,82]. However, comprehensive study on the vulnerability and resilience of the karst ecosystem is still in the blank state. Therefore, the development of a research framework that is universally applicable to the combination of vulnerability and resilience of karst ecosystems is a question that needs to be addressed in future research.

#### 4.2.2. There Is no Uniform Standard for the Selection of Vulnerability Assessment Indicator of Village Ecosystem: Appropriate Assessment Indicator System Should Be Constructed According to the Natural Geographical Background Characteristics of Villages and the Threat Factors Faced by Village Ecosystem

The characteristics of the village ecosystem in different regions are different, and the disturbance factors of the village ecosystem are also different. In the existing village ecosystem vulnerability research, the indicator selection has not reached unity. Therefore, it is necessary to clarify the characteristics of village ecosystems in different regions and build a suitable evaluation indicator system. The main sensitive source and exposure source of the ecosystem in the rocky desertification control area come from the comprehensive effect of the local natural environment and human activities. The natural environment is a background source; human activities have caused pressure on the environment and accelerated and promoted this process. Therefore, when constructing the indicator system, it is necessary to construct a scientific and reasonable evaluation indicator system according to the natural and human particularity of the village ecosystem, which is one of the problems that need to be paid attention to in the evaluation of ecosystem vulnerability of rocky desertification control villages.

#### 4.2.3. In View of the Problems of Different Levels of Rocky Desertification Control Village Ecosystem Vulnerability, Unclear Vulnerability Factors and Lack of Research, we should Strengthen the Rocky Desertification Control Village Ecosystem Vulnerability and its Influencing Factors

The article *Sustainability science*, published in *Science* in 2001, makes clear that the question of what factors determine the fragility and resilience of a particular type of ecosystem is at the heart of the science of sustainable development [83]. The karst environment is one of the most fragile ecological zones in the world. Because of its special natural environment and strong karst effect, the karst ecosystem is fragile and sensitive, which is easily disturbed by the outside world and leads to ecological destruction, and it is difficult to restore after destruction. Due to the unreasonable social and economic activities of human beings, the vegetation is destroyed, soil erosion and land productivity decline, and the surface of the earth presents a gradual evolution process of rock bareness similar to the desert landscape [84]. However, the vulnerability and influencing factors of the special village ecosystem formed by governance are still unclear, and the research is still in a vacant state. Therefore, it is necessary to strengthen the research on the vulnerability of the ecosystem and its influencing factors in rocky desertification control villages, so as to provide scientific and technological references for the implementation of control strategies and the improvement of ecosystem services in villages.

#### 4.2.4. In View of the Lack of Research on the Control Strategy of Ecosystem Vulnerability in Karst Rocky Desertification Control Villages, the Control Strategy of Ecosystem Vulnerability in Karst Rocky Desertification Control Villages Should Be Discussed on the Basis of Vulnerability Assessment, so as to Provide Scientific Reference for Rocky Desertification Control

The study of ecosystem vulnerability is helpful to understand the impact of natural factors and human factors on the ecosystem, and has scientific guiding significance for the governance of ecosystem vulnerability and the improvement of ecosystem services. At present, aiming at the problem of ecosystem vulnerability in karst areas, Guo et al. studied the spatiotemporal change pattern and driving mechanism of ecosystem vulnerability in the karst mountainous areas of southwestern China [80]. Chen et al. studied the difference of vulnerability between karst nature reserves and non-karst nature reserves and its influencing factors [85]. However, there are some deficiencies in the study of ecosystem vulnerability and its control strategies, especially for the village ecosystem formed by rocky desertification control. Therefore, it is necessary to strengthen the research on the control strategy of the village ecosystem vulnerability in rocky desertification control, so as to provide scientific reference for further rocky desertification control.

#### 4.2.5. In View of the Weak Application of Village Ecosystem Resilience Research in Practice, we should Combine the Theory of Resilience Research with Practical Application, and Give Full Play to the Practical Significance of Resilience Research

The current measurement of resilience faces conceptual and methodological obstacles [86]. The research on village ecosystem resilience theory is the precondition for developing the resilience strategy, and it has an important guiding role in the management of the ecosystem. Theoretical research should be applied to practice in order to improve the practicability of research results. This is especially the case for the rocky desertification control ecosystem, due to the special natural background; the environmental water retention is poor and the depression easily accumulates water. Rocky desertification areas are prone to seasonal drought, floods, landslides and debris flows and other natural disasters because of rugged terrain, shallow soil and weak soil reinforcement capacity, as well as climate factors. The education level of village community residents is relatively low, the knowledge of disaster response is lacking, the reserve of production and living materials is not much, and the livelihood diversity is poor, which causes the rocky desertification villages to have a lack of resilience strategies and weak recovery ability. Therefore, it is necessary to strengthen the combination of theory and practical application of ecosystem resilience research, improve the resilience of the rocky desertification control village ecosystem, and promote the sustainable development of the ecosystem.

#### 4.2.6. In View of the Lack of Research on Ecosystem Resilience Enhancement Strategies for Rocky Desertification Control, the Research on Resilience Enhancement Strategies Should Be Strengthened According to the Ecosystem Resilience Level and Driving Factors of Rocky Desertification Control

Ecological management and restoration in ecologically fragile areas has always been a hot issue of international concern, and researchers from all over the world have been committed to exploring the best path of sustainable development in ecologically fragile areas. In the study of ecologically fragile area management, the coordination of regional development and ecological protection and restoration is very important to achieve regional sustainable development. At present, how to coordinate the development of the ecological environment and social economy is a common concern. Resilience is an inherent property of the system itself, and its theoretical study is of great importance in guiding ecosystems to improve their ability to cope with disturbances and achieve sustainable development. The ecosystem formed by rocky desertification control provides services such as carbon sink, environmental purification, oxygen supply and water conservation, in addition to daily production and livelihood. However, the level and driving factors of ecosystem resilience in rocky desertification control are still unknown. There is a lack of strategies to enhance the resilience of rocky desertification control ecosystems. Therefore, it is necessary to strengthen the study of ecosystem resilience strategies for rocky desertification control. In addition, we must provide a theoretical reference for promoting sustainable development of the rocky desertification control ecosystem.

#### 4.2.7. The Innovation of Research Methods and Technical Means Needs to Be Strengthened, and Multi-Disciplinary and Multi-Technical Means should Be Used to Analyze the Vulnerability and Resilience of Ecosystems

Research methods and technical means are the basic path of quantitative research on fragile human–land systems and practical activities. There are many research methods in the development of ecosystem vulnerability research. Because different system elements have different impacts on the ecosystem, the relationship between system elements will also have an impact on the ecosystem, and these impacts are manifested in different aspects; the complexity of the ecosystem is far more complex than that described by the model, and there is great uncertainty in the quantitative and qualitative research on ecosystem vulnerability [87]. Therefore, in order to enhance the accuracy of the evaluation results, we should adhere to scientific and rational principles, integrate multi-disciplinary methods and use multi-technical means to comprehensively analyze the vulnerability characteristics of regional ecosystems.

#### 4.2.8. In View of the Different Characteristics and Driving Factors of the Vulnerability of Rocky Desertification Ecosystem at Different Times, the Time Scope of the Study Should Be Further Expanded

The driving factors of ecosystem vulnerability are different in different periods, and the degree of vulnerability is also different. Ecological vulnerability assessment results are time-sensitive. The diversification of research tools and methods is useful. Scholars apply 3S technology to vulnerability assessment; it achieves the evaluation of temporal evolution and is no longer confined to static evaluation. However, comparative studies on the vulnerability characteristics of rocky desertification control ecosystems at different time periods are still inadequate. The changing characteristics of the degree of vulnerability and drivers before and after ecological management and at different stages of management are more indicative of the state of vulnerability and the mechanisms of evolution at each stage. This provides greater insight into the key drivers of change in vulnerability at each stage. Extending the time scale of the study is an important guide to identify the main vulnerability factors in the process of rocky desertification control and to develop targeted management measures.

## 5. Conclusions and Future Research

In this paper, the current research progress on vulnerability and resilience of the village ecosystem was analyzed through systematic literature review. The research on vulnerability and resilience of the village ecosystem is accelerating: the study areas are mainly in the coastal countries of Asia, North America and Africa, and most of them are in China, the United States and India; most of the research literatures are published by research institutions in China, the United States, India and Australia, and the research units are mainly universities and research institutes (centers); the research focuses on index system, monitoring and evaluation, influencing factors and strategy research. Based on the analysis of the research status and progress, eight key scientific and technological problems to be solved are put forward, which point out the direction for further research in the future.

According to the existing research literature, the current village ecosystem vulnerability and resilience research has not yet formed a perfect theoretical system. Theoretical and methodological research needs to be further expanded and deepened; empirical research is mostly quantitative evaluation; quantitative research methods are not mature; empirical research uses existing conceptual models to build the index system; there is a lack of innovation and lack of pertinence. The study of vulnerability and resilience of the village ecosystem need to be further strengthened in the following aspects: (1) to strengthen the theoretical and methodological research on vulnerability and resilience of the village ecosystem, one must deepen the connotation research and construct a scientific and rational conceptual model combining vulnerability and resilience of the village ecosystem. (2) Disturbance factors of different types of village ecosystems are different, and the interaction of system elements is different, which leads to the unique formation mechanism of vulnerability and resilience of village ecosystems, so the question of how to reveal the formation mechanism of vulnerability and resilience of different types of village ecosystems is the key scientific problem to be solved in this field. (3) There is no mature quantitative research framework and method for vulnerability and resilience of the village ecosystem, and the existing evaluation methods through the index system need to be further expanded and deepened in determining weight and selecting indicators. (4) The development of village ecosystem vulnerability and resilience governance practice lags behind the empirical research of case sites, so it is necessary to strengthen the connection between vulnerability and resilience theoretical research and governance practice. (5) The village ecosystem is constantly affected by natural and manmade disturbance factors; its resilience is affected by the interaction of many factors. The resilience mechanism of the village ecosystem is complex. Therefore, it is necessary to strengthen the research on the resilience mechanism of the village ecosystem. (6) The village ecosystem is a complex comprehensive system composed of human society and the natural environment. This system is constantly developing and changing in the process of operation. How to reveal the resilience dynamics of the village ecosystem and realize the resilience process deduction and prediction are the problems that need to be solved in future research. How to solve the above scientific problems and address technological needs in the study of rocky desertification control village ecosystem is the problem to be solved and the challenge to be faced in future research.

## Data Availability

Data are contained within the article.
